# Histone-modifying enzymes as drivers and therapeutic targets in androgen-resistant prostate cancer

**DOI:** 10.3389/fendo.2025.1730397

**Published:** 2026-01-12

**Authors:** Tanaya A. Purohit, Kayla Bahr, Bing Yang, Zachery Schultz, Peter W. Lewis, John M. Denu, David F. Jarrard

**Affiliations:** 1 Department of Urology, School of Medicine and Public Health, University of Wisconsin, Madison, WI, United States; 2 Cancer Biology Program, University of Wisconsin, Madison, WI, United States; 3 Carbone Comprehensive Cancer Center, University of Wisconsin, Madison, WI, United States; 4 Department of Biomolecular Chemistry, University of Wisconsin, Madison, WI, United States; 5 Wisconsin Institute for Discovery and the Morgridge Institute for Research, University of Wisconsin, Madison, WI, United States

**Keywords:** androgen resistance, castration-resistant prostate cancer, epigenetics (chromatin remodeling), EZH2 overexpression, histone acetylation, histone methylation, NSD2, prostate cancer

## Abstract

Androgen deprivation therapy (ADT) remains the cornerstone of treatment for advanced, hormone-sensitive prostate cancer (HSPC), but responses are transient, and most patients ultimately develop castration-resistant prostate cancer (CRPC), a largely incurable stage of disease. The mechanisms driving resistance are not yet fully understood. Recent data suggest epigenetic dysregulation driven by alterations in chromatin remodelers and histone-modifying enzymes (HMEs) contributes significantly to prostate cancer (PC) progression and resistance to androgen-directed therapies. HMEs control chromatin structure and transcriptional programs, and their altered activity contributes to androgen resistance and tumor progression. HME inhibitors offer promising therapeutic potential, yet their effects are highly context-dependent, emphasizing the importance of biomarker-guided precision strategies and rational combination therapies. This review highlights the contribution of histone PTMs and HMEs to CRPC progression and discusses their potential as novel strategies to improve clinical outcomes.

## 1 Introduction

Prostate cancer (PC) is the second most commonly diagnosed malignancy, and the fifth leading cause of cancer-related deaths among males worldwide, with an estimated 1.5 million cases annually (1). Primary PC is dependent on androgens and androgen receptor (AR) signaling to survive and proliferate (2). Since the landmark discovery by Huggins and Hodges in 1941, demonstrating the hormonal sensitivity of PC cells, androgen deprivation therapy (ADT) has been the cornerstone treatment for advanced, metastatic hormone-sensitive prostate cancer (HSPC) (3). Metastatic HSPC patients treated with ADT eventually progress to castration-resistant prostate cancer (CRPC), with a median survival of approximately 3 years, and it remains incurable. Combination approaches using ADT with AR signaling inhibitors (ARSI), chemotherapy have improved outcomes by targeting advanced HSPC (3). For example, large phase 3 clinical trials (CHAARTED, LATITUDE, and STAMPEDE) have found that combining docetaxel or abiraterone, an ARSI, with ADT significantly prolongs survival in metastatic HSPC patients (4–6). Nonetheless, therapy resistance inevitably develops, underscoring the need to identify molecular drivers of this transition to CRPC and novel therapeutic targets enabling personalized treatment strategies.

Molecular alterations such as *ETS* gene fusions, *SPOP*, *FOXA1*, and *IDH1* mutations define primary PC subtypes that ultimately contribute to metastasis (2). The most common molecular subtype, *ERG* fusion-positive (46%), is driven by *ERG-TMPRSS2* gene fusion, which leads to overexpression of the *ERG* oncogene and altered AR signaling. It usually co-occurs with loss of *PTEN* (25%) and *TP53* (8%), driving metastasis (2, 7). Usually, *SPOP* (11%) and *FOXA1* (3%) mutations are essential genomic drivers of the non-*ETS* subtype, along with *CHD1* loss (15%) and alterations in DNA damage repair genes promoting progression to metastatic PC (2). Metastatic tumors accumulate AR pathway and MYC amplifications, loss of *RB1* and *TP53* (25%), PI3K pathway activation, and develop androgen resistance, reflecting strong selective pressures from ADT and/or ARSIs (8). The transition to androgen resistance is an area of intense interest, given the benefit of combining active therapies (9, 10). In CRPC, AR signaling and its downstream targets are commonly overexpressed due to gene mutations, splice variants, or amplification of the *AR* gene (11). AR signaling also relies on a network of coactivators that amplify its transcriptional activity and drive tumor progression, particularly after ADT. For example, DNA repair proteins like PARP1 and TOP2B can function as AR coactivators, linking AR signaling to genomic instability (12, 13). Together, these studies demonstrate that progression to androgen resistance is driven by genetic alterations, therapy selection by AR amplification, DNA repair deficiency, and chromosomal instability.

Epigenetic dysregulation is emerging as a central feature of PC progression and therapy resistance (14, 15). Histone post-translational modifications (PTMs), regulated by histone-modifying enzymes (HMEs), alter chromatin architecture and gene expression and are frequently disrupted in cancer (16–18). Other than their canonical function of catalyzing different histone PTM reactions, they can function as AR transcriptional coactivators and promote DNA damage repair, regulate PI3K/AKT, WNT signaling, and enhance tumor angiogenesis (19). Nuclear receptor coactivators, including the SRC family (NCOA1–3), can further potentiate AR target gene expression (20), while chromatin regulators such as BRD4, MED1, and CHD1 coordinate transcriptional output (21, 22). Recent research has also identified key HMEs such as P300/CBP and TIP60 (acetyltransferases), and EZH2 and NSD2 (methyltransferases), that enhance AR signaling by remodeling chromatin and activating oncogenic transcriptional programs, further contributing to CRPC development (23–25).

Evolving data have demonstrated differing HME patterns in CRPC adenocarcinomas compared to neuroendocrine and other atypical AR-independent PCs that can arise after treatment with ARSIs. CRPC adenocarcinomas typically retain dependence and expression of the AR and exhibit elevated activity of AR-target genes. In contrast, neuroendocrine prostate cancer (NEPC) displays altered epigenetic reprogramming marked by loss of AR activity and a shift toward HMEs that drive lineage plasticity and neural differentiation. EZH2 is markedly upregulated in NEPC, where it represses AR-target genes through regulating H3K27me3 and cooperates with SOX2 and BRN2 to generate a neuroendocrine state (26). NEPC may also rely more heavily on class I HDACs and sirtuins such as SIRT1 and SIRT7, which promote dedifferentiation, transcriptional silencing, and tumor aggressiveness (27). This developing area suggests that NEPC and atypical cancers have chromatin landscapes that may benefit from HME-targeted therapies and highlight opportunities to exploit differential HME dependencies for precision treatment.

This review summarizes the biology of histone PTMs and HMEs, their roles in the development of androgen resistance, and the current status of HME inhibitors in preclinical and clinical development. We also discuss how genetic alterations shape the chromatin landscape in CRPC and their potential utility as predictive biomarkers for precision therapy. In addition, recent developments and limitations of therapeutic agents targeting HMEs in PC are discussed.

## 2 Histone post-translational modifications and histone-modifying enzymes

In 1942, C.H. Waddington coined the term “epigenetics” to describe the complex, dynamic interactions between genes and their products that lead to the development of a phenotype. Epigenetic modifications commonly include DNA methylation, histone PTMs, microRNAs, and long non-coding RNAs to regulate gene expression (19). Various modifications act in a concordant fashion to regulate chromatin structure and influence gene expression. Of these, DNA methylation and histone PTMs are key epigenetic processes that modulate and organize chromatin looping, which becomes dysregulated in cancer (28, 29). DNA methylation has been intensively investigated, while the field of histone PTMs and HMEs is a newer area of research.

**Figure 1:**
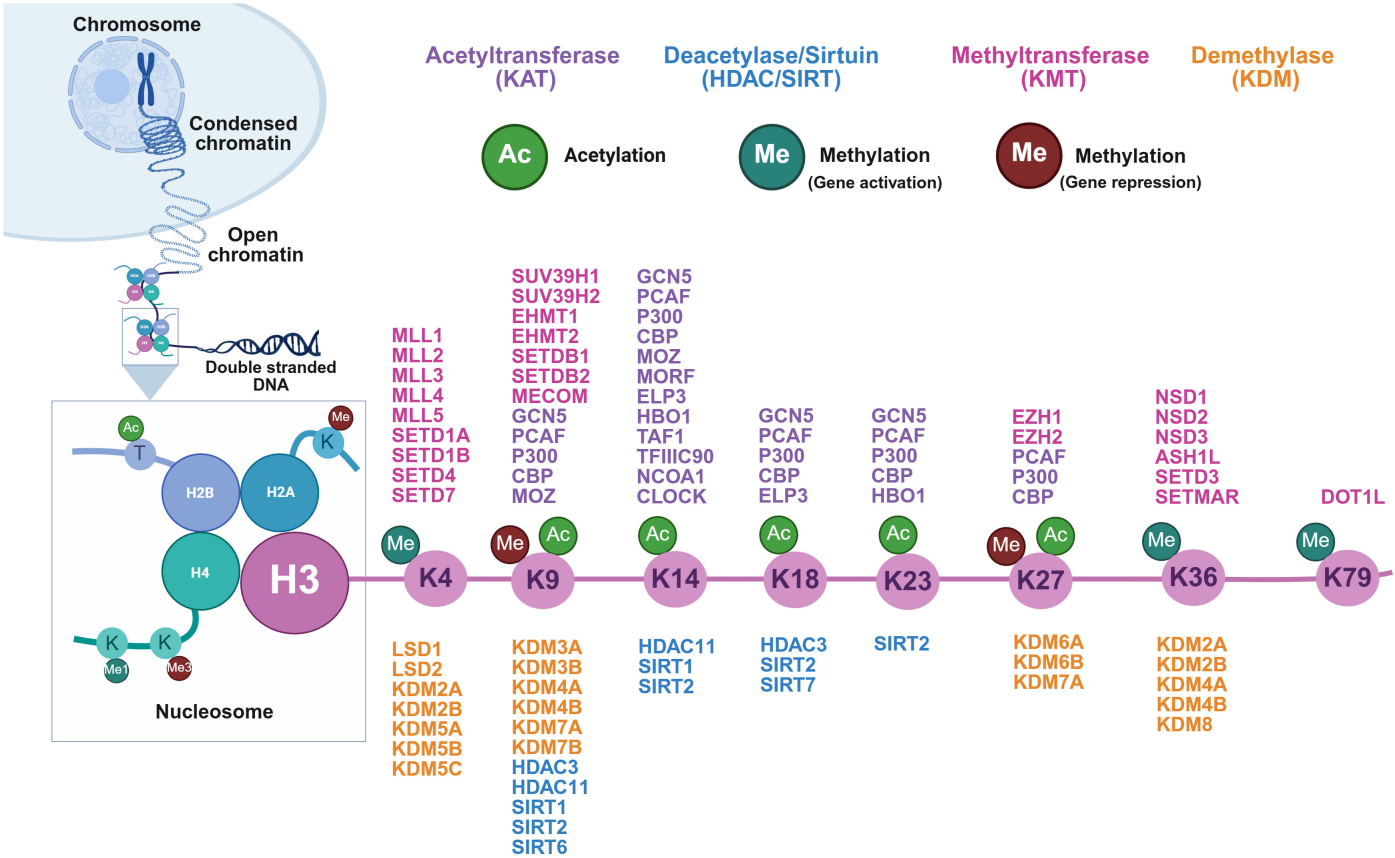
Histone-modifying enzymes (HMEs) for histone H3 lysine post-translational modifications (PTMs), such as acetylation and methylation. Chromosomes are condensed chromatin structures compared to DNA wrapped around histone octamers (H2A, H2B, H3, and H4) to form nucleosomes. The N-terminal tails can undergo a variety of PTMs, including acetylation and methylation. HMEs catalyze these modifications at specific amino acid residues, a mechanism that modulates chromatin structure and gene expression. This schematic highlights all known HMEs that regulate acetylation and methylation at specific lysine (K) residues: 4, 9, 14, 18, 23, 27, 36, and 79, on histone H3 protein, which have been implicated in prostate tumorigenesis.

Histone proteins (H2A, H2B, H3, and H4) are highly conserved and form a histone octamer (nucleosome), the basic unit of DNA packaging to form chromosomes (**Figure 1**). Histone N-terminal tail amino acid residues undergo various PTMs such as methylation, acetylation, phosphorylation, SUMOylation, ubiquitylation, ADP ribosylation, citrullination, and biotinylation at specific amino acid residues (19). Histone H3 is further categorized into canonical histones H3.1 and H3.2 that are uniformly deposited genome-wide. At the same time, the non-canonical H3.3 variant is preferentially incorporated into actively transcribing regions (30). HMEs that regulate acetylation and methylation of specific lysine (K) residues on histone H3 protein are of active interest as targets in prostate tumorigenesis (**Figure 1**).

**Figure 2:**
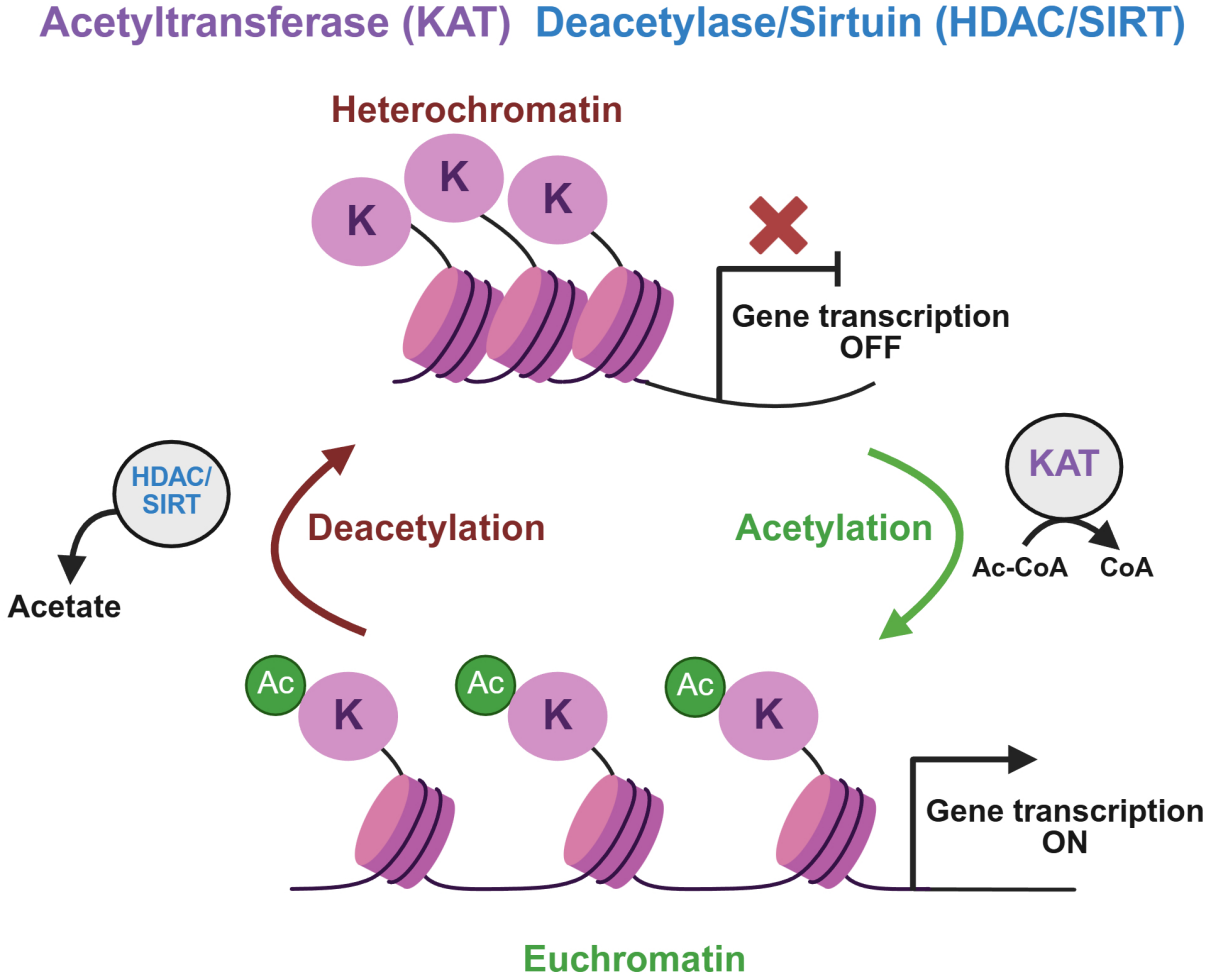
Histone acetylation and deacetylation reactions. The schematic illustrates histone H3 lysine (K) acetylation and deacetylation processes. Acetylation of H3 lysine residues is mediated by lysine acetyltransferases (KAT) via the conversion of acetyl coenzyme A (Ac-CoA) to coenzyme A (CoA). This modification is associated with an open chromatin architecture (euchromatin) and transcriptional activation. The process is reversible through histone deacetylase (HDAC) and sirtuin (SIRT) activity, which restores a condensed chromatin state (heterochromatin). This structural compaction inhibits transcription factor and RNA machinery binding at promoter and enhancer regions, resulting in gene silencing. Reprogramming of chromatin acetylation patterns occurs, including H3K27ac, with progression to castration-resistant disease, and includes increased P300/CBP activity and decreased SIRT2 expression. Overexpression of HDACs can occur and may contribute to aberrant deacetylation of histones and non-histone proteins, potentially repressing tumor suppressor genes or altering chromatin states that promote malignant transformation and progression.

A group of enzymes called lysine acetyltransferases (KAT) carries out acetylation at the target lysine (K) residue on histone H3 protein. In the acetylation reaction, acetyl coenzyme A (here Ac-CoA) is converted into coenzyme A (CoA). Histone acetylation results in more relaxed euchromatin formation, promoting active gene transcription (**Figure 2**) (19). They are divided into five super families based on sequence and structural similarities and functions: (1) GNAT (GCN5, PCAF and ELP3), (2) P300/CBP, (3) MYST (TIP60, MOZ, MORF, HBO1 and MOF), (4) Cytoplasmic HAT (HAT1 and 4), and (5) transcriptional coactivators (TAF1, TFIIC90, NCOA and CLOCK). The histone acetylation reaction is reversible by the removal of the acetyl group from lysine residues. This reaction is carried out by histone deacetylases (HDAC) and sirtuins (SIRT) (**Figure 2**). They are divided into four classes: (1) Class I (HDAC1–3 and 8), (2) Class II (HDAC4–7 and 9-10), (3) Class III (SIRT1-7), and (4) Class IV (HDAC11) (31). Histone deacetylation results in more compact chromatin structures, resulting in gene repression (**Figure 2**) (32).

**Figure 3:**
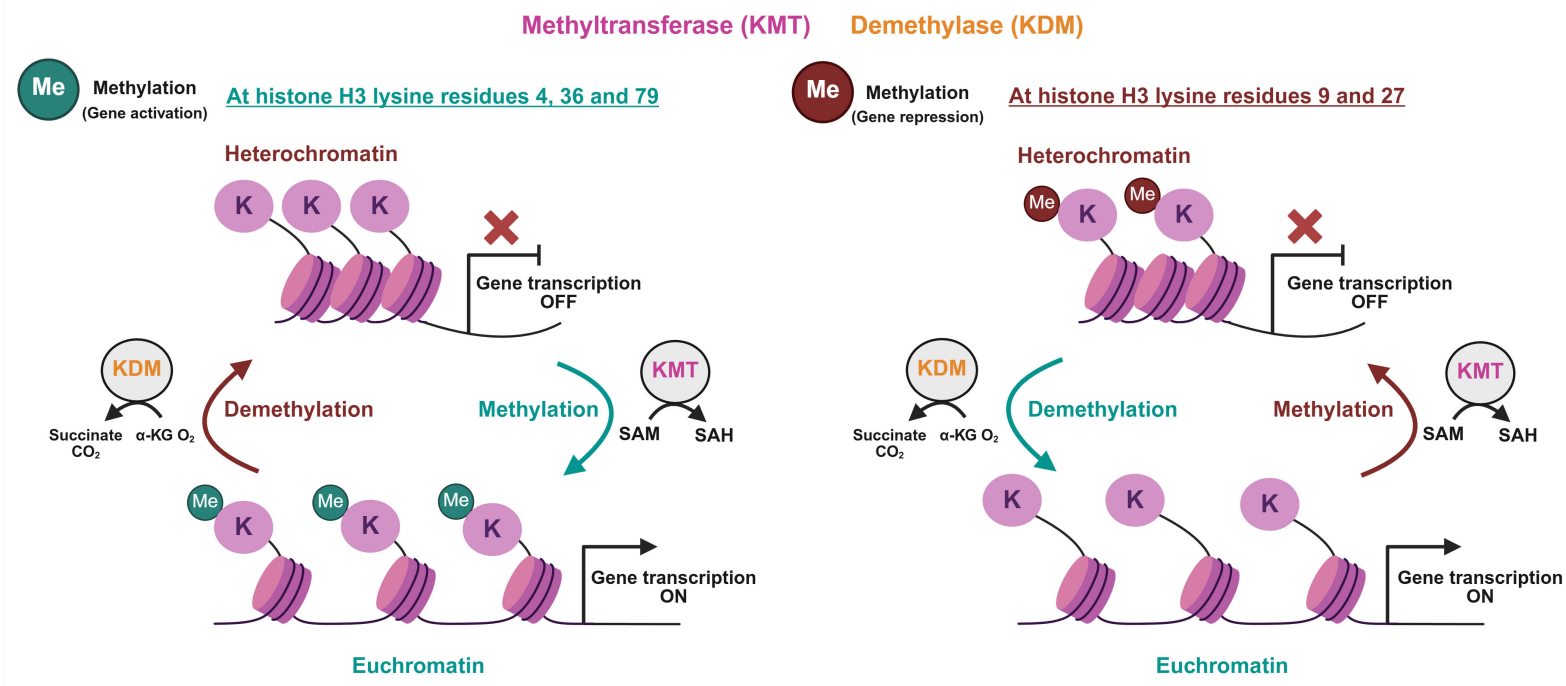
Histone methylation and demethylation reactions. The schematic illustrates histone H3 lysine (K) methylation and demethylation, major epigenetic processes that contribute to the development of androgen resistance. Histone methylation is catalyzed by histone methyltransferases (KMT), utilizing S-adenosylmethionine (SAM) as a methyl donor to yield mono-, di-, or tri-methylated lysines and S-adenosylhomocysteine (SAH) as a byproduct. This process is reversible through oxidative reactions mediated by histone demethylases (KDM). The specific lysine residue and methylation state determine transcriptional outcomes. Methylation at H3K4, H3K36, and H3K79 (specifically H3K4me3, H3K36me2, and H3K79me2/3) is associated with euchromatin formation and transcriptional activation; removal of these methyl groups leads to gene silencing. Conversely, trimethylation of H3K9 and H3K27 results in heterochromatin formation and the exclusion of transcriptional machinery, leading to gene repression, while demethylation at these sites restores chromatin accessibility. KMTs and KDMs shift the histone methylation balance in ways that support AR signaling reactivation or bypass, enable survival under ADT, and metastatic traits.

Similar to histone acetylation, histone methylation adds a methyl group from the donor S-adenosylmethionine (SAM) to different H3 lysine (K) residues, converting SAM into S-adenosylhomocysteine (SAH) (**Figure 3**) (19). Lysine residues can be either mono, di, or tri-methylated (31). HMEs that catalyze the addition and removal of methyl groups are termed lysine methyltransferases (KMT) and demethylases (KDM), respectively. KMTs can be grouped based on the lysine residue they target; however, KDMs can demethylate lysine at different positions (**Figure 3**). Except for DOT1L, the rest of the KMTs possess the catalytic SET domain. The effect of histone methylation is complex and much more context-dependent, leading to either gene expression or repression depending on the target lysine and methylation state (31). Methylation of H3K4, H3K36, and H3K79 is generally associated with transcriptionally active euchromatin, while methylation of H3K9 and H3K27 is associated with transcriptionally repressed, compacted heterochromatin (**Figure 3**) (31).

**Figure 4:**
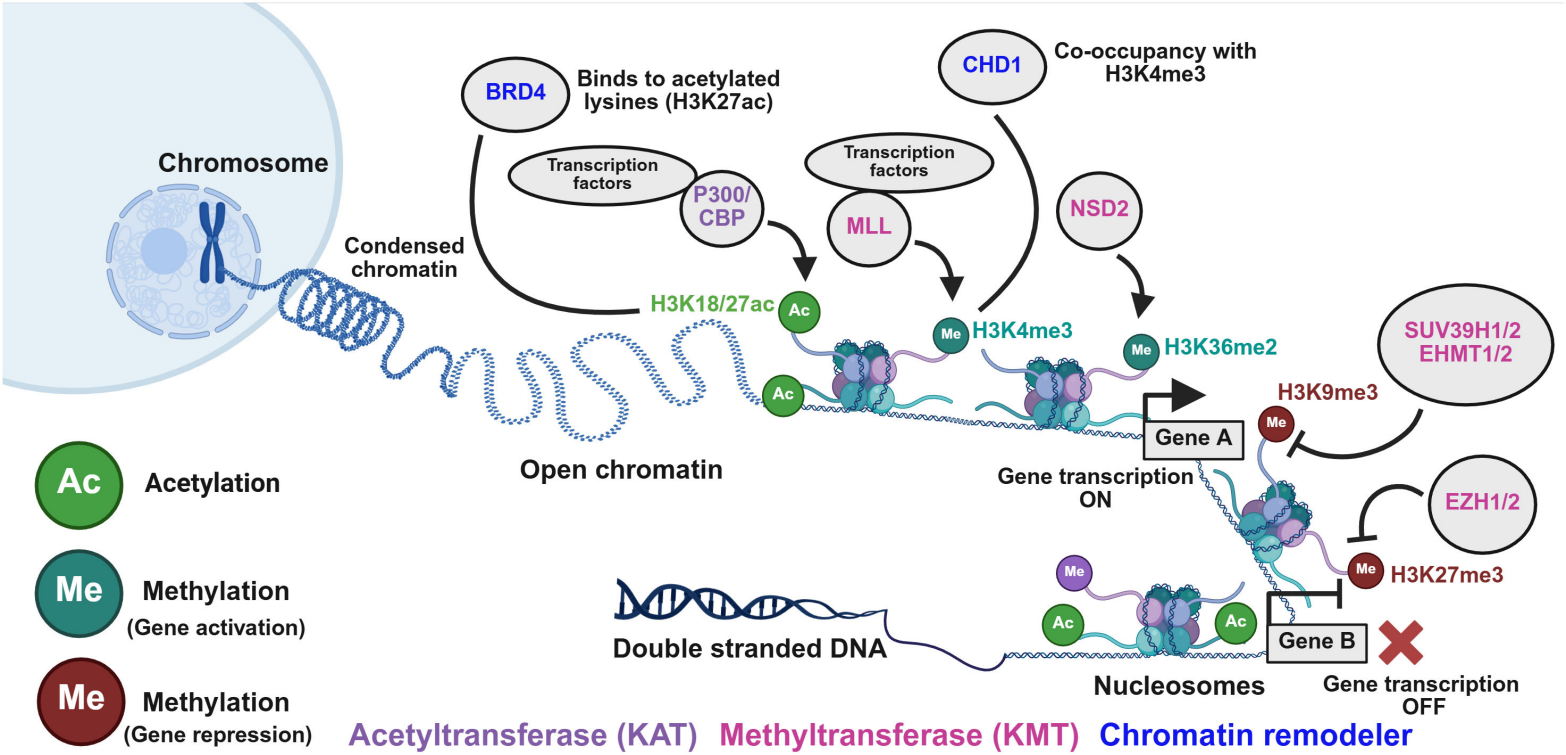
Regulation of histone acetylation and methylation by various histone-modifying enzymes (HMEs) and chromatin remodelers. Dynamic regulation of histone acetylation and methylation by HMEs and chromatin remodelers. KATs (P300/CBP) and KMTs (MLL and NSD2) catalyze the addition of acetyl (H3K27ac) and methyl (H3K4me3 and H3K36me2) groups, respectively. Chromatin remodelers, including BRD4 and CHD1, play a critical role in recognizing the histone marks associated with gene activation and are dysregulated in some PC cases. They help recruit transcription factors and other regulatory proteins to gene promoters and enhancers. Conversely, processes like deacetylation and the establishment of repressive methylation marks (H3K27me3 and H3K9me3) by KMTs such as EZH1/2 and SUV39H1/2 lead to chromatin compaction and ultimately result in silencing of genes involved in tumor suppression.

The levels of histone acetylation and methylation are tightly regulated by a dynamic equilibrium between the expression and activities of several HMEs (**Figure 4**). KATs (P300/CBP) and KMTs (NSD2, MLL, EZH2) add acetyl and methyl groups, respectively, while HDACs and SIRTs remove acetyl groups, and KDMs erase methyl marks. Histone PTMs do not act independently; instead, they occur in a highly specific and combinatorial manner by the action of several HMEs and chromatin remodelers (BRD4, CHD1). This in turn, promotes the recruitment of transcription factors and other regulatory proteins at promoters and enhancer regions (**Figure 4**) (32). Together, this network of histone PTMs and HMEs, with chromatin remodelers, acts as a regulatory system that controls chromatin accessibility. Thus, further characterization of their roles in promoting PC progression and the development of castration resistance would allow for the discovery of novel and potent targeted therapies.

## 3 Dysregulation of histone acetylation with the development of castration resistant prostate cancer

Acetylation of histone proteins is one of the first identified PTM that promotes active gene transcription. Dysregulation of histone acetylation and its associated enzymes contributes to cancer initiation, development, and progression. Growing evidence has also highlighted a role for histone acetylation in advanced PCs (32–35).

Recent work highlights an important role acetylation plays in the development of androgen independence. Using a high-throughput peptide microarray assay, our research group found global alterations in histone acetylation after CRPC development. C4–2 cells, an isogenic CRPC derivative of LNCaP (HSPC), displayed increased acetylation at H3K9, H3K14, and H3K18, driven by elevated KAT P300 activity and reduced SIRT2 deacetylase expression (32). Enhanced P300 activity resulted from autoacetylation at lysine residue 1499. Increased H3K18ac was further validated in CRPC xenografts, patient tissue samples, and circulating tumor cells (32, 34). Together, these data show that heightened P300 activity and diminished SIRT2 reshape histone H3 acetylation patterns in CRPC. Other than histone H3 acetylation, P300 and CBP also catalyze H2B N-terminal acetylation, which is a key mark for active enhancers and gene promoters. H2B acetylation facilitates the formation of enhanceosomes, which are complexes that assemble on enhancers to drive the transcription of key oncogenes such as *MYC* and *CCND1* in PC (36). Thus, targeting histone H2B acetylation and the associated enhanceosome formation via P300/CBP degradation represents a novel and promising strategy for disrupting the epigenetic regulation in PC.

Histone acetylation changes also hold clinical significance. In patient tissue microarrays, H3K18ac and P300 were elevated in primary and metastatic PC, whereas SIRT2 progressively declined (33). Increased P300 associated with aggressive pathologic features, and reduced SIRT2 predicted adverse clinical outcomes, including PSA recurrence. In the SU2C/PCF CRPC dataset, low SIRT2 expression was linked to shorter time to progression on enzalutamide or abiraterone, underscoring the relevance of the P300–SIRT2–H3K18ac axis in CRPC (34).

Beyond enzymatic activity, P300/CBP also functions as a critical coactivator for AR-mediated transcription in PC, stabilizing AR protein and promoting acetylation at AR target loci such as *KLK3*, in cooperation with steroid receptor coactivators (25). The deacetylase SIRT7 was also found to be essential in maintaining the oncogenic phenotype by selective deacetylation of H3K18 (37). In other solid tumors, SIRT7 is highly enriched at promoters of tumor suppressor genes such as nucleoside diphosphate kinase A (*NME1*), and COP9 signalosome complex subunit 2 (*COPS2*), as well as the ribosomal protein genes, to inhibit their transcription and promote cell growth and invasion (38). Lesser characterized HMEs, including GCN5, PCAF, HDAC2, and HDAC5, are overexpressed in CRPC and have been linked to poor prognosis and PSA recurrence (32). Collectively, these findings underscore altered histone acetylation as a critical driver of CRPC biology and a potential therapeutic vulnerability.

## 4 Genetic alterations lead to dysregulation of histone post-translational modifications with CRPC development

Molecular analyses identify unique subtypes of PC, generating interest in developing subtype-specific biomarkers and therapies has increased (7). While the accumulation of genetic alterations during progression to androgen resistance is well recognized, these changes remain difficult to directly target with pharmacologic agents. In contrast, dysregulation of HMEs represents a potentially reversible vulnerability through selective enzyme inhibition. Recent work from our research group demonstrated that primary PCs harboring *CHD1* deletion acquire a unique histone PTM landscape with the transition to CRPC, characterized by reduced H3.3K27K36 methylation (39). *CHD1*, a chromatin remodeler that regulates nucleosome assembly, euchromatin accessibility, and transcriptional activity, is recurrently deleted in approximately 13–17% of primary PC cases (40, 41). Loss of *CHD1* is associated with reduced H3.3K36me2 levels and downregulation of the KMT NSD2. Importantly, *CHD1*-functional CRPC tumors exhibit heightened sensitivity to NSD2 inhibition, suggesting a biomarker-driven therapeutic opportunity to exploit *CHD1*-specific epigenetic vulnerabilities (42).

Alterations in other chromatin remodeling complexes, particularly the SWI/SNF complex, further contribute to PC progression and androgen resistance (43, 44). Loss or mutation of key SWI/SNF subunits, including *ARID1A*, *SMARCA2/4*, and *PBRM1*, is linked to genomic instability, aberrant chromatin accessibility, and dysregulated gene expression (42, 45). Preclinical studies indicate that inhibition of SWI/SNF function can enhance susceptibility to chemotherapy, immunotherapy, and PARP inhibitors (42, 45). Together, these findings highlight chromatin remodelers as both biomarkers of disease aggressiveness and potential therapeutic targets.

## 5 Histone lysine methyltransferases (KMT) in prostate cancer

Aberrant expression of KMTs disrupts histone PTMs, altering chromatin accessibility and transcriptional programs. KMTs are highly selective for the histone lysine residue that they target, and they function within multiprotein complexes (46). Several KMTs have emerged as central drivers of PC progression and therapeutic targets in CRPC.

### 5.1 EZH2

EZH2 is one of the core subunits of the multiprotein complex Polycomb Repressive Complex 2 (PRC2), which includes SUZ12 and EED, both essential for complex stability and activity (47). EZH2 catalyzes trimethylation of histone H3 on lysine 27 (H3K27me3), leading to heterochromatin formation and transcriptional silencing (47). SUZ12 and EED are essential to maintain PRC2 complex stability and EZH2 catalytic activity. First identified as an altered KMT in prostate tumorigenesis, EZH2 is now recognized as a critical regulator of cancer initiation, metastasis, and resistance to ADT (48). Elevated EZH2 expression is consistently observed in metastatic HSPC and CRPC compared to benign and primary prostate tissue, and is associated with poor clinical outcomes, including increased recurrence risk, tumor aggressiveness, and reduced survival (48, 49). Overexpression of EZH2 is also a recurrent feature across many malignancies, such as bladder, breast, lung, liver, and hematologic cancers, reinforcing its broad oncogenic role and therapeutic relevance (50, 51).

In PC, canonically, EZH2-mediated histone H3K27 trimethylation represses and silences developmental and tumor suppressor genes, driving proliferation, invasion, angiogenesis, and therapy resistance (52). EZH2 is transcriptionally activated by the ERG oncogene, an ETS family member transcription factor, which is frequently overexpressed in primary PC due to the AR-regulated *ERG–TMPRSS2* gene fusion (53). ERG-driven EZH2 upregulation promotes epigenetic reprogramming and the activation of oncogenic pathways, including IGFR, MEK, WNT, NFκB, and PI3K/AKT, that facilitate tumor progression and metastasis (52, 53).

Chinaranagari and colleagues demonstrated the functional crosstalk between H3K27me3 and DNA methylation in PC progression (54). The transcriptional silencing of the tumor suppressor gene *ID4* is mediated through overexpression of EZH2 and increased deposition of H3K27me3 at the ID4 promoter, creating a repressive histone environment that facilitates recruitment of DNA methyltransferase DNMT1. This, in turn, leads to *de novo* promoter DNA methylation, locking ID4 into a stably silenced state, providing one of the most direct mechanistic links between histone PTMs and DNA methylation in PC progression (54). Both mechanisms underscore the multilayered epigenetic architecture that governs tumor suppressor silencing and contributes to prostate tumorigenesis. Combination treatment with a DNA demethylating agent such as 5-aza-2′-deoxycytidine (5-aza-dC) and an EZH2 inhibitor, GSK126, induced re-expression of tumor suppressor genes such as *IGFBP7* and *SFRP1*, and showed an inhibitory effect on PC cell growth *in vitro* (55).

EZH2 functions as both a methyltransferase enzyme and a transcriptional cofactor. EZH2 mediates methylation of non-histone proteins such as FOXA1, enhancing its stability by blocking ubiquitin-specific protease 7 (USP7). Stabilized FOXA1 promotes expression of cell-cycle genes and proliferation, and dual inhibition of EZH2 and USP7 markedly suppresses tumor growth in FOXA1-high PCs *in vitro* and *in vivo* (56). Another role of EZH2 in CRPC occurs independently of the PRC2 complex. This involves phosphorylation at serine 21 (via PI3K/AKT signaling), which converts EZH2 into a transcriptional coactivator that directly interacts with AR to drive AR-target gene expression (23). This dual functionality underscores why pharmacologic inhibition of EZH2 offers therapeutic promise, blocking both its canonical methyltransferase activity and its non-canonical role as an AR coactivator.

### 5.2 NSD2

NSD2, a KMT responsible for di-methylation of histone H3 lysine 36 (H3K36me2), has emerged as an exciting oncogenic driver and therapeutic target in CRPC. H3K36me2 accumulates on active gene bodies and promotes open chromatin conformation and gene transcription (57). NSD2 levels are elevated in patients with primary and metastatic PC, correlating with higher Gleason scores and poor patient prognosis, and are significantly increased with the development of CRPC (24, 49, 58). Overexpression of NSD2 has also been documented in multiple solid tumors, including breast, lung, renal, and gastric cancers, and is frequently amplified in multiple myeloma due to the *t(4;14)* translocation, where the *NSD2* gene is under the control of the *IgH* promoter (59). Together, these observations underscore NSD2 as a bona fide oncogene and therapeutic target across several malignancies.

Mechanistically, NSD2 interacts with the DNA-binding domain of AR via its HMG box domain, enhancing AR activity and transcription of target genes such as *KLK3* and *NKX3.1 in vitro* in LNCaP cells (60). Using a CRISPR screen approach, Parolia and colleagues identified NSD2 as an essential coactivator of AR signaling in LNCaP cells (24). NSD2 and its H3K36 methyltransferase activity recruit AR to tumor-specific enhancers, where it cooperates with pioneer factors such as FOXA1, HOXB13, and ETS, redistributing AR binding and amplifying oncogenic signaling (24). This suggests that NSD2 is a critical coactivator of AR in CRPC that enables its oncogenic activity.

Beyond AR signaling, NSD2 contributes to metastasis through multiple pathways, such as DNA damage repair, epithelial to mesenchymal transition (EMT), PI3K/AKT, WNT, and NFkB signaling (59). For example, NSD2 enhances NFkB signaling by directly interacting with activated NFkB to promote transcription of downstream pro-survival genes such as *IL6*, *CCND1*, and *BCL2*, thereby promoting tumor growth (61). The expression of AKT is elevated in PTEN-deficient PC, which promotes NSD2 protein stability. Mechanistically, AKT phosphorylates NSD2 and prevents its degradation by CRL4^Cdt2^ E3 ligase. Stabilized NSD2 transcriptionally upregulates *RICTOR*, a key subunit of the mTOR pathway, further driving metastasis (62). NSD2-mediated deposition of H3K36me2 at the *TWIST* gene locus similarly promotes EMT and progression (63). Aytes and colleagues also report using murine models that NSD2 is a key driver of metastasis with enrichment of pathways associated with metastatic progression, such as EMT, E2F, MYC, TGFβ, and p53 signaling (58). Moreover, NSD2 can methylate non-histone substrates, including PTEN and TIAM1, thereby enhancing DNA repair and PI3K/AKT signaling (64, 65).

Finally, a regulatory axis between EZH2-NSD2 has been described, mediated by a network of microRNAs, including miR-203, miR-26, and miR-3 in PC (66). EZH2-driven H3K27me3 silences these microRNAs, thereby stabilizing NSD2 expression and elevating H3K36me2, which promotes tumor growth and metastasis. Collectively, these findings position NSD2 as a central oncogenic regulator of chromatin state, AR signaling, and metastatic progression in PC. Importantly, emerging small-molecule NSD2 inhibitors, currently under preclinical investigation, provide a promising therapeutic avenue to exploit these vulnerabilities (67). Further development and clinical translation of such agents could offer new precision treatment options for patients with CRPC.

### 5.3 Other KMTs

Expanding evidence suggests other KMTs play a role in the transition to CRPC. For example, MLL1, an H3K4 KMT and driver of MLL fusion-positive leukemia, also functions as a coactivator of AR, promoting the growth of CRPC cells (68). The multiprotein MLL1 complex directly interacts with AR via its scaffold subunit menin, a 67kDa protein frequently overexpressed in CRPC and associated with poor clinical outcomes (68, 69). Targeting menin to disrupt MLL1–AR interactions has shown promising antitumor activity in preclinical CRPC models, highlighting this axis as a potential therapeutic vulnerability.

Another class of KMTs, including SUV39H1/2 and EHMT1/2, which catalyze histone H3 lysine 9 methylation (H3K9me), is also upregulated during prostate tumorigenesis (70, 71). EHMT1 expression is induced in enzalutamide-treated LNCaP cells, leading to the accumulation of H3K9me3, contributing to drug resistance and immune evasion (72). In parallel, EHMT2 has been implicated in promoting bone metastasis through activation of the PI3K/mTOR pathway (73, 74). Despite the oncogenic potential of these enzymes, efforts to target them remain limited, representing an underexplored area of drug discovery and development.

Lastly, DOT1L, the sole H3K79 methyltransferase containing a DOT1-like enzymatic domain, is reported to be overexpressed in PC and is associated with poor clinical outcomes (75, 76). Mechanistically, DOT1L stabilizes AR and MYC protein stability in HSPC, potentially playing a role in resistance to ADT (76). Inhibiting DOT1L impairs PC tumor growth and colony formation by suppressing AR and MYC signaling in preclinical models (76). These findings suggest that a combination of DOT1L inhibitors with ADT may be a future viable therapeutic strategy for HSPC.

## 6 Histone lysine demethylases (KDM) in prostate cancer

KDMs are characterized by their ability to remove methyl groups from histone lysine residues, resulting in either gene expression or repression (46). These enzymes modulate AR through a number of approaches, but also directly alter metabolic enzymes and oncogenic pathways. One of the more extensively studied KDMs in PC is KDM1A (LSD1), an enzyme that usually demethylates histone H3K4 to maintain gene repression in normal tissue. However, Metzger and colleagues observed that LSD1 switches function by removing repressive histone marks on H3K9 to activate AR-target genes (77). In CRPC xenografts, LSD1 is overexpressed and promotes multiple oncogenic pathways such as MYC, E2F, and FOXA1 signaling (78). Independent of its demethylase function, recent work demonstrates that LSD1 and its binding protein ZNF217 activate a pro-survival gene network in CRPC cells (79). Inhibiting LSD1 induces apoptosis and reduces cell growth, providing a rationale for ongoing trials targeting LSD1 in PC alone or in combination (**Table 1**).

**Table 1:** HME inhibitors in clinical testing for CRPC.

Target	Inhibitor name	Mechanism of action	Study design	Clinical trial ID	Intervention	Status and main findings	Ref
P300/CBP+BET	EP31670	Inhibits P300/CBP and BET proteins	Phase 1 Study	NCT05488548	Monotherapy	Ongoing	(91)
P300/CBP	Inobrodib	Inhibits the bromodomain of P300/CBP	Phase 1/2a Open-Label Study	NCT03568656	Inobrodib and Abiraterone or Enzalutamide or Darolutamide or Olaparib or Atezolizumab	Completed: Results not published yet	(91)
P300/CBP	Pocenbrodib	Blocks P300/CBP and inhibits the acetylation of histone and non-histone proteins	Phase 1b/2a, Multicenter, Open-Label Study	NCT06785636	Pocenbrodib and Abiraterone or Olaparib or 177Lu-PSMA-617	Not yet recruiting	(91)
HDAC	Panobinostat	Non-selective pan-HDAC inhibitor	Phase 1/2 Study	NCT00878436, NCT00667862, NCT00663832, NCT00493766, NCT00670553, NCT00419536	Panobinostat and Bicalutamide or Docetaxel and Prednisone or External Beam Radiotherapy	Panobinostat alone did not show any clinical activity. However, when combined with bicalutamide, it extended progression-free survival by 6 months. Combination of panobinostat with docetaxel resulted in a partial response, with a>50% decline in PSA levels, but increased toxicities were observed, with no relevant anti-tumor activity. The results from the panobinostat and external beam radiotherapy study have not been published.	(92, 93)
HDAC	Vorinostat	Broad inhibitor of HDAC activity and inhibits class I and class II HDACs	Phase 1/2 Study	NCT06145633, NCT00330161, NCT01174199, NCT00589472, NCT00565227, NCT00045006, NCT00005634	Vorinostat and 177Lu-PSMA-617 or mTOR inhibitor Temsirolimus or docetaxel or neoajuvant ADT followed by Radical Prostatectomy	7% patients did not progress at 6 months after treatment with vorinostat alone. The combination therapy studies with the mTOR inhibitor and docetaxel were terminated due to no efficacy and increased toxicity.	(94)
HDAC	Pracinostat	Selective HDAC inhibitor for class I, II, and IV	Phase 2 Study	NCT01075308	Monotherapy	Pracinostat alone did not show sufficient activity based on the PSA response rate to further investigate it as a single agent for CRPC.	(95)
HDAC	Belinostat	Inhibits HDAC activity	Phase 1 Study	NCT00413322, NCT04703920, NCT00413075	Belinostat and 5-Fluorouracil or Talazoparib	A combination of Belinostat and Talazoparib exhibits a favorable safety profile and manageable toxicity; however, further studies are needed to determine the efficacy of this combination. The results from the Belinostat and 5-Fluorouracil study are not available.	(96)
HDAC	Entinostat	Selective HDAC inhibitor for class I and IV	Phase 1 Study	NCT00020579, NCT03829930	Entinostat and Enzalutamide	Entinostat alone resulted in biologically relevant antitumor activity. However, the sponsor discontinued the drug, and the combination study with enzalutamide was terminated.	(97)
HDAC	Romidespsin	Non-selective pan-HDAC inhibitor	Phase 1/2 Study	NCT00106301, NCT00106418, NCT01638533	Monotherapy	Romidepsin demonstrated minimal antitumor activity; however, further studies are needed to improve HDAC inhibition alone or in combination with other therapies.	(98)
HDAC	Mecetinostat	Inhibits HDAC1/2/3/11 activity	Phase 1, Open-Label, Dose-Escalation Study	NCT00511576	Mecetinostat and Docetaxel	Trial terminated because Celgene ended its collaboration agreement with MethylGene for the development of mecetinostat.	(91)
EZH2	GSK2816126	SAM competitive inhibitor that reduces H3K27me3 levels	Phase 1 Open-Label, Dose Escalation Study	NCT02082977	Monotherapy	The study was terminated due to a lack of antitumor clinical activity at maximum tolerated doses.	(100)
EZH2	Tazemetostat	SAM competitive inhibitor that reduces H3K27me3 levels	Phase 1b/2 Open-Label Study	NCT04179864	Tazemetostat and Enzalutamide or Abiraterone/Prednisone	The combination therapy extended progression-free survival. However, it was not statistically significant, and the study was terminated.	(101)
EZH2	CPI-1205	SAM competitive inhibitor that reduces H3K27me3 levels	Phase 1b/2 Study	NCT03480646	CPI-1205 and Enzalutamide or Abiraterone/Prednisone	The study was terminated due to a lack of an improved response rate with the combination therapy.	(91)
EZH2	Mevrometostat	Inhibits EZH2 activity and reduces H3K27me3 levels	Phase 1, Dose Escalation Study; Phase 3 Randomized, Double Blind and Open-Label Study	NCT03460977; NCT06629779, NCT06551324	Mevrometostat and Enzalutamide or Docetaxel	The combination therapy with enzalutamide shows promising clinical activity.	(9)
EZH2	MAK683	Selectively binds to the EED subunit of the PRC2 complex that inhibits EZH2 catalytic activity.	Phase 1/2, Multicenter, Open-Label Study	NCT02900651	Monotherapy	MAK683 alone showed limited clinical activity as a single agent in CRPC patients.	(103)
EZH2	SHR2554	Oral, small-molecule inhibitor exhibiting potent selectivity for EZH2.	Phase 1/2 Open-Label Study	NCT03741712	SH2554 and Rezvilutamide	The study was terminated due to unknown reasons.	(91)
EZH2	XNW5004	Selectively inhibits EZH2 activity and reduces H3K27me3 levels	Phase 1b/2 Open-Label Study	NCT06022757; NCT06702995	XNW5004 and Enzalutamide/Pembrolizumab	Ongoing	(91)
EZH1/2	CPI-0209	Dual EZH1/2 inhibitor that reduces H3K27me3 levels	Phase 1/2 Study	NCT04104776	Monotherapy	Ongoing	(91)
EZH1/2	Valemetostat	Dual EZH1/2 inhibitor that reduces H3K27me3 levels	Phase 1 Study	NCT04388852	Valemetostat and Ipilimumab	Ongoing	(91)
NSD2	KTX-2001	Potent and selective oral inhibitor of NSD2	Phase 1, Dose Escalation Study	NCT07103018	KTX-2001 and Darolutamide	Ongoing	(91)
LSD1	CC-90011	Potent, selective, and reversible oral inhibitor of LSD1	Phase 1, Open-label Study	NCT04628988	CC-90011 with Abiraterone and Prednisone	Completed: Results not published yet	(91)
LSD1, HDAC6	JBI-802	Selectively inhibits LSD1 and HDAC6 within the CoREST complex	Phase 1/2, Open-label, Dose Escalation and Expansion Study	NCT05268666	Monotherapy	Ongoing	(91)

List of clinical trials that are completed or are in progress to test the efficacy of small-molecule inhibitors against P300/CBP, HDAC, EZH1/2, NSD2, and LSD1 alone or in combination with other existing therapies for the treatment of CRPC.

Several additional demethylases within the KDM3 and KDM4 families are overexpressed in prostate tumor progression and play a variety of roles in the development of androgen resistance. For example, a key coactivator of the AR is KDM3A (JMJD1A), which removes repressive H3K9 marks, facilitating AR binding and generating active AR-V7 splice variants involved in ADT resistance (80). It also enhances DNA repair through MYC pathways, creating treatment resistance to radiation therapy and PARP inhibitors (81, 82). KDM3B regulates key amino acids and metabolic enzymes, including arginase 2 and retinol dehydrogenase 11, supporting CRPC growth (83). Similar to LSD1, KDM4B contributes to AR transcriptional activity by demethylating H3K9 (84). KDM4B also acts as an AR co-activator, can induce AR-V7 splicing in CRPC, and its overexpression confers resistance (85). Recent data indicate that inhibition of KDM4B reduces global levels of both H3K9me3 and H3K27me3, resensitizing CRPC cells to the AR pathway inhibitor enzalutamide and inhibiting tumor growth (84).

Emphasizing differing demethylase functions, KDM5D, which is located on the Y chromosome, acts as a tumor suppressor, and its loss leads to dysregulated AR signaling and resistance to ADT/taxane combinations in a large trial (86). KDM6A overexpression promotes AR signaling, contributing to ADT resistance, whereas KDM6B is required for the transcriptional upregulation of oncogenic genes such as *MYC*, cyclin D1, and *RB1* (87). Finally, PHF8 (KDM7B) acts as an AR co-activator, undergoes activation in hypoxia, and promotes metastasis by increasing cell migration and invasion (88, 89). Collectively, these studies provide proof-of-principle that targeting KDMs may represent an effective epigenetic therapeutic strategy in CRPC.

## 7 Therapeutic strategies to target HMEs and limitations

**Figure 5:**
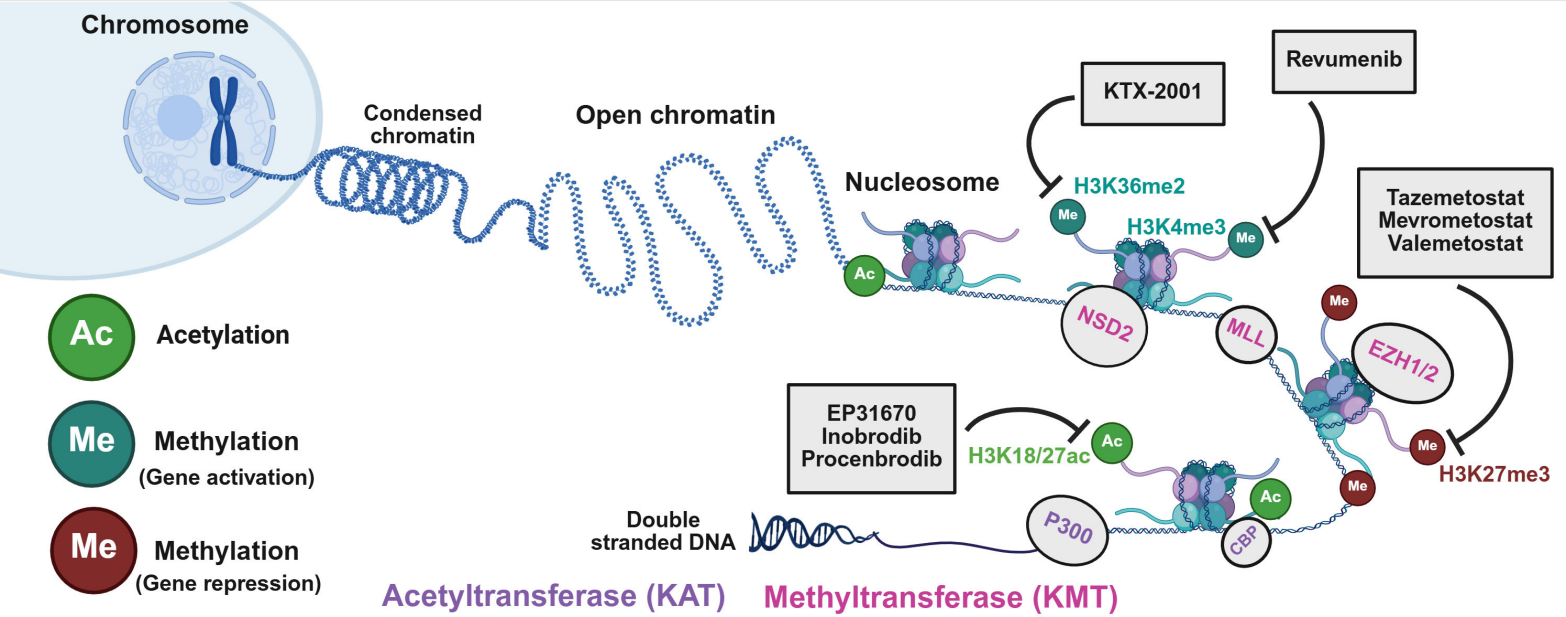
Histone-modifying enzyme (HME) inhibitors in clinical trials as monotherapy or combined with other treatments for metastatic PC and CRPC patients. Schematic representation of major small-molecule inhibitors specific for histone H3 acetylation and methylation. KATs (P300/CBP) and KMTs (MLL and NSD2) catalyze the addition of acetyl (H3K27ac) and methyl (H3K4me3 and H3K36me2) groups, respectively. Inhibiting these modifications attenuates transcription of oncogenic programs. Whereas inhibiting the deposition of H3K27me3 marks by EZH1/2 results in activation of tumor suppressor genes. Several targeted inhibitors, such as EP31670, Inobrodib, Procenbrodib (KAT inhibitors), and KTX-2001, Revumenib, Tazemetostat, Mevrometostat, Valemetostat (KMT inhibitors), are shown to disrupt their respective enzymatic activities. Collectively, these HMEs and their pharmacological modulators shape chromatin accessibility and transcriptional regulation in the development of PC progression to an androgen-resistant state.

Current therapeutic strategies for PC across different clinical stages include surgery, ADT and/or ARSIs, and chemotherapy. While these approaches are effective for managing advanced disease, resistance inevitably emerges. Given the critical role of HMEs in PC initiation and progression, there has been growing interest in targeting HMEs as novel epigenetic therapies (**Figure 5**). Ongoing clinical trials are investigating inhibitors of P300/CBP, HDAC, EZH2, NSD2, and LSD1 in hematologic malignancies and solid tumors, including CRPC (**Table 1**).

P300/CBP functions as a transcriptional coactivator with both enzymatic acetyltransferase activity and a bromodomain that recognizes acetylated lysine on histones (90). Preclinical studies have identified potent inhibitors that disrupt these functions, leading to suppression of oncogenic transcription (90). Clinical trials are ongoing to test the safety and efficacy of inhibitors such as EP31670, inobrodib, and pocenbrodib (**Figure 5**) in metastatic CRPC or in combination with other therapies, including ARSIs, olaparib, radiopharmaceutical therapy, and immunotherapy (NCT05488548, NCT03568656, NCT06785636) (91). Given the multifaceted biological role of P300/CBP in prostate tumorigenesis, many clinical studies are anticipated in the future.

HDACs are overexpressed in PC and other cancers, and their inhibition has shown promise in hematologic malignancies with agents such as panobinostat (92, 93), vorinostat (94), pracinostat (95), belinostat (96), entinostat (97), romidepsin (98), and mecetinostat (91). However, in CRPC, HDAC inhibitors have yielded modest clinical success, often accompanied by significant toxicity and disease progression. This limited efficacy may be due to the context-dependent and sometimes opposing roles of different HDACs, as well as the lack of HDAC-specific inhibitors. As a result, combination therapies are being explored to enhance their therapeutic benefit (99).

EZH2, the catalytic component of the PRC2 complex, mediates transcriptional repression through H3K27 trimethylation. Preclinical models show that EZH2 inhibition can induce apoptosis, suppress proliferation, and reduce H3K27me3 levels (23, 48, 56, 66). GSK126 (GSK2816126) is a highly selective and potent SAM competitive inhibitor of EZH2 and one of the first small-molecule inhibitors of EZH2 to enter clinical trials in hematologic malignancies and solid tumors. Early clinical trials with GSK126 demonstrated limited efficacy in PC (NCT02082977) (100). Next-generation compounds like tazemetostat (NCT04179864) (**Figure 5**) and CPI-1205 (NCT03480646) showed limited clinical activity alone as well as when combined with enzalutamide for CRPC (101). More potent mevrometostat, a selective, orally bioavailable small molecule inhibitor of EZH2, is under active investigation alone or in combination with enzalutamide and docetaxel in CRPC (NCT03460977, NCT06629779, NCT06551324) (**Figure 5**) (9, 91). Importantly, compensatory activity by EZH1 has been observed following EZH2 inhibition (50, 51), prompting the development of dual EZH1/2 inhibitors such as CPI-0209 and valemetostat (91). Valemetostat is currently being investigated in combination with the immune checkpoint inhibitor ipilimumab for metastatic PC (NCT04388852) (**Figure 5**) (102). Other strategies include targeting the EED subunit of PRC2 (MAK683; NCT02900651), although clinical activity has been limited in CRPC (103). To date, tazemetostat is the only EZH2 inhibitor approved by the FDA for epithelioid sarcoma and follicular lymphoma (104).

NSD2, an H3K36 methyltransferase, promotes PC cell proliferation, migration, and metastasis (62, 66). Preclinical studies suggest that inhibiting NSD2 reduces proliferation, colony formation, and induces apoptosis. Several small-molecule inhibitors and PROteolysis TArgeting Chimeras (PROTACs) have been developed, including UNC8153, UNC8723, and LLC0424, though most have had limited success in preclinical studies due to a lack of potency and structural homology with other H3K36 enzymes, resulting in poor specificity for NSD2 (67). To overcome this, researchers are developing strategies to target PWWP1 and PHD domains that are unique to NSD2. Efforts include the development of UNC8153 and its second-generation degrader UNC8723 for multiple myeloma (105, 106) and LLC0424 that reduce H3K36me2 levels along with cell growth suppression *in vitro* and *in vivo* (107). In August 2022, KTX-1001, a potent and selective NSD2 inhibitor that binds to the SET domain, developed by K36 Therapeutics, Inc., entered a phase 1 clinical trial for multiple myeloma (NCT05651932), marking the first NSD2-targeting agent for cancer treatment, raising hope for future drug development in PC (67). There is a lack of detailed literature available regarding KTX-1001’s structure and preclinical data. Recently, the FDA granted clearance to the phase 1 STRIKE-001 trial (NCT07103018) evaluating KTX-2001, an oral inhibitor of NSD2, for patients with metastatic CRPC as monotherapy and in combination with darolutamide, a second-generation ARSI (**Figure 5**) (91).

Beyond these major enzymes, several other epigenetic regulators are under investigation. Inhibitors of the MLL1–menin interaction, such as MI-136 and MI-503, have shown the ability to suppress AR signaling and PC growth in preclinical CRPC models (108). Recently FDA approved Revumenib, an oral small-molecule inhibitor of the MLL1-menin complex, for the treatment of relapsed/refractory acute leukemia (**Figure 5**) (109). DOT1L inhibitors such as EPZ004777 and EPZ5676 that employ competitive SAM inhibition strategies impair tumor growth in PC models (76), with EPZ5676 advancing to clinical trials in leukemia (110). LSD1 inhibitors such as CC-90011 (NCT04628988) and dual inhibitors like JBI-802 that target both LSD1 and HDAC6 (NCT05268666) are currently in clinical testing for advanced solid tumors, including CRPC, intending to reverse androgen resistance and restore sensitivity to AR-directed therapies (111, 112). Together, these studies highlight the therapeutic potential of targeting HMEs in PC but also underscore the complexity of their biological roles.

Limitations to the application of HME-targeted therapies remain significant. Continued investigation will refine inhibition strategies to improve selectivity and identify effective combinations with existing treatments. The identification of biomarkers will be important for guiding trial design and patient selection. Differential HME expression in advanced PC, such as overexpression of NSD2 or P300, will be important to define therapeutic windows (32, 49). Approximately a third of circulating tumor cells in CRPC identified as adenocarcinomas demonstrate elevated acetylated P300 (34). Preclinical studies also suggest that HME inhibitors can resensitize resistant tumors when used in combination regimens. For example, NSD2 inhibition restores androgen receptor expression and re-sensitizes neuroendocrine tumors to enzalutamide (113).

Toxicity and off-target effects pose major barriers to HME inhibitor clinical development. Dose-limiting toxicities, including hematologic and gastrointestinal issues, and fatigue and gastrointestinal are well-documented (93, 100). Many HMEs belong to large enzyme families that contain overlapping catalytic and substrate specificity, complicating selective targeting and enabling compensatory activity by other family members, a challenge highlighted in HDAC inhibition clinical inhibitor studies (94, 98). For example, panobinostat did not show sufficient clinical activity due to pharmacological limitations, failure with PSA decline, and unmanageable toxicities in CRPC patients (93). Moreover, HMEs can regulate numerous non-histone substrates involved in essential processes such as metabolism, DNA repair, and signal transduction, increasing the risk of toxicity (65). These limitations underscore the need for more selective agents, improved biomarkers for target engagement, and rational combination strategies to fully harness HME inhibitors as effective cancer therapeutics.

The biological consequences of HME inhibition are highly context-dependent. Because HMEs regulate fundamental processes such as chromatin accessibility, transcriptional regulation, and DNA repair, their inhibition can suppress oncogenic pathways in certain settings while inadvertently impairing anti-tumorigenic programs or promoting therapy resistance in others. For example, EZH2 inhibitors show potent antitumor activity in lymphomas with EZH2 gain-of-function mutations (104), yet in some NEPC models, EZH2 blockade did not promote lineage reversal back to an AR-driven luminal sensitive state, but rather a forward differentiation to a more terminal ARSI-resistant neuroendocrine state (26). Similarly, LSD1 inhibitors can resensitize CRPC to AR blockade, but in lung adenocarcinoma, LSD1 inhibition disrupts EGFR and KRAS pathways with variable effects on tumor growth (79, 114). In the case of HDAC inhibitors, although they have demonstrated efficacy in hematologic malignancies such as cutaneous T-cell lymphoma but their use in solid tumors has often been limited by toxicity and context-specific resistance mechanisms (99). These examples illustrate that the biological and clinical outcomes of HME-targeted therapies are highly context-dependent, influenced by cancer type, stage, molecular subtype, tumor-specific molecular drivers, and compensatory chromatin regulatory mechanisms.

## 8 Conclusions and future directions

Epigenetic dysregulation due to alterations in chromatin remodelers and HMEs contributes substantially to PC progression and therapy resistance. ADT has been recognized as the standard approach for managing advanced PC for over 75 years. Despite longstanding efforts to target the AR signaling axis in PC, resistance remains a major hurdle. Inhibition of HMEs has emerged as a promising therapeutic strategy in PC, but its effects are highly context-dependent and can lead to either beneficial or detrimental outcomes, with the outcomes dependent on tumor subtype, other molecular drivers, and chromatin state. This highlights how much remains to be learned about the interplay between chromatin regulation and tumor evolution. It also emphasizes the need for precision approaches guided by molecular biomarkers, patient stratification, and combination therapy to maximize treatment benefit and minimize unintended consequences.

Looking ahead, HMEs represent an exciting frontier for next-generation PC therapies, particularly for advanced and treatment-resistant disease. As the epigenetic mechanisms driving CRPC progression become increasingly well defined, this will enable the development of more selective and potent inhibitors. Recent advances in multi-omic technologies, such as CUT&RUN, CUT&Tag, whole-genome bisulfite sequencing, along with ChIP-seq, or array-based platforms, have greatly enhanced our ability to map histone PTMs, DNA methylation, chromatin accessibility, and HMEs in PC (115). Integrating comprehensive genomic, transcriptomic, and epigenomic profiling at the single-cell level into clinical trial design will be critical for identifying biomarkers that predict therapeutic response and monitor target engagement. Advances in drug design will facilitate the creation of inhibitors that more precisely target catalytic domains or disrupt oncogenic protein–protein interactions within HME complexes. A major application of these drugs will involve combination strategies that leverage HME inhibition to overcome lineage plasticity, reverse AR-independent phenotypes, or resensitize tumors to AR-directed therapies. As the field progresses, rationally designed HME-based therapies guided by precision medicine frameworks will provide new opportunities to improve clinical outcomes for patients with PC.
